# Case report: Autoimmune glial fibrillary acidic protein astrocytopathy with overlapping autoimmune syndrome

**DOI:** 10.3389/fimmu.2024.1485374

**Published:** 2024-10-11

**Authors:** Wu-xiao Wei, Ming-li Chen, Lian Meng

**Affiliations:** Guangxi University of Science and Technology First Affiliated Hospital, Liuzhou, China

**Keywords:** anti-GFAP antibody, aquaporin-4 antibody, NMDAR antibody, autoimmune glial fibrillary acidic protein astrocytopathy, central nervous system

## Abstract

Autoimmune glial fibrillary acidic protein (GFAP) astrocytopathy is a rare autoimmune disease, which is characterized by the immune system attacking astrocytes in the central nervous system, resulting in inflammation and damage to the nervous system. We reported a 41-year-old female patient with only drowsiness for 3 months, who was, otherwise, healthy with no other signs of meningoencephalitis or myelitis. There were no obvious abnormalities in her neurological and ophthalmic tests. Brain magnetic resonance imaging (MRI) plain scan + enhancement with the gadolinium contrast agent revealed patchy hypointensity on T1-weighted imaging, hyperintensity on T2-weighted imaging, hyperintensity on T2-weighted fluid-attenuated inversion recovery in the left basal ganglia, corona radiata, and local septum pellucida, with no enhancement in the enhanced lesions. Cerebrospinal fluid (CSF) revealed white blood cell count of 5.00 × 10^6^/L, CSF protein of 828.53 mg/L, and glucose of 2.83 mmol/L. Aquaporin-4 (AQP4) antibody, N-methyl-D-aspartate receptor (NMDAR) antibody and GFAP antibody were all positive, whereas the remaining autoimmune encephalitis antibody tests were negative. Oncology screening [including head, chest, and whole-abdomen (involving the pelvic cavity) CT and tumor markers] did not reveal any obvious evidence of tumor presence. The patient received systemic treatment with high-dose intravenous injection of steroids combined with immunosuppressive agents, and the clinical and imaging features of the patients improved. To the best of our knowledge, reports on overlapping positivity of AQP4 antibody and NMDAR antibody in patients with GFAP astrocytopathy were still very rare. We hope to supplement the existing literature on this topic, review the relevant literature, and strive to increase the understanding toward GFAP astrocytopathy with overlapping autoimmune syndrome so as to enable early diagnosis and early treatment and to improve the clinical outcome of patients.

## Introduction

Autoimmune glial fibrillary acidic protein (GFAP) astrocytopathy is a novel inflammatory autoimmune disease of the central nervous system (1, 3). The clinical manifestation of GFAP astrocytopathy is complex; moreover, it can coexist with a variety of antibodies, including aquaporin-4 (AQP4) antibody and N-methyl-D-aspartate receptor (NMDAR) antibody and is, therefore, called overlap syndrome (2). In this study, we presented a case of a patient with GFAP astrocytopathy with overlapping autoimmune syndrome, who was admitted and treated in our hospital. The patient received a long course of steroid combined with immunosuppressive agent treatment and achieved remarkable therapeutic effect.

In this case study, emphasizing the rarity of GFAP astrocytopathy combined with overlap syndromes aims to enhance clinical awareness among healthcare professionals. By providing detailed descriptions of the case, etiology, clinical manifestations, and diagnostic methods, it helps improve physicians’ ability to recognize such rare conditions, facilitating timely and accurate diagnosis and treatment. Such reports play a crucial role in expanding physicians’ knowledge base and increasing alertness to rare diseases.

## Case report

A 41-year-old female patient residing in Liuzhou City, Guangxi Zhuang Autonomous Region, visited our hospital and was admitted as a result of “drowsiness for more than 3 months.” Her family complained that the patient experienced excessive daytime drowsiness without an obvious cause 3 months ago, with daytime sleep lasting more than 12 h. The symptoms persist without significant improvement. She denied a history of respiratory and digestive tract infections in the past 3 months; a history of chronic diseases such as “heart disease, hypertension, and kidney disease”; a history of infectious diseases including “tuberculosis, typhoid fever, dysentery, and viral hepatitis”; and a history of food and drug allergies. There were no obvious abnormalities in her neurological examinations, including time and place orientation, language assessment, cranial nerves, ophthalmic testing, movement, sensation, coordination, and gait. At the same time, her physical examination was normal.

**Figure 1:**
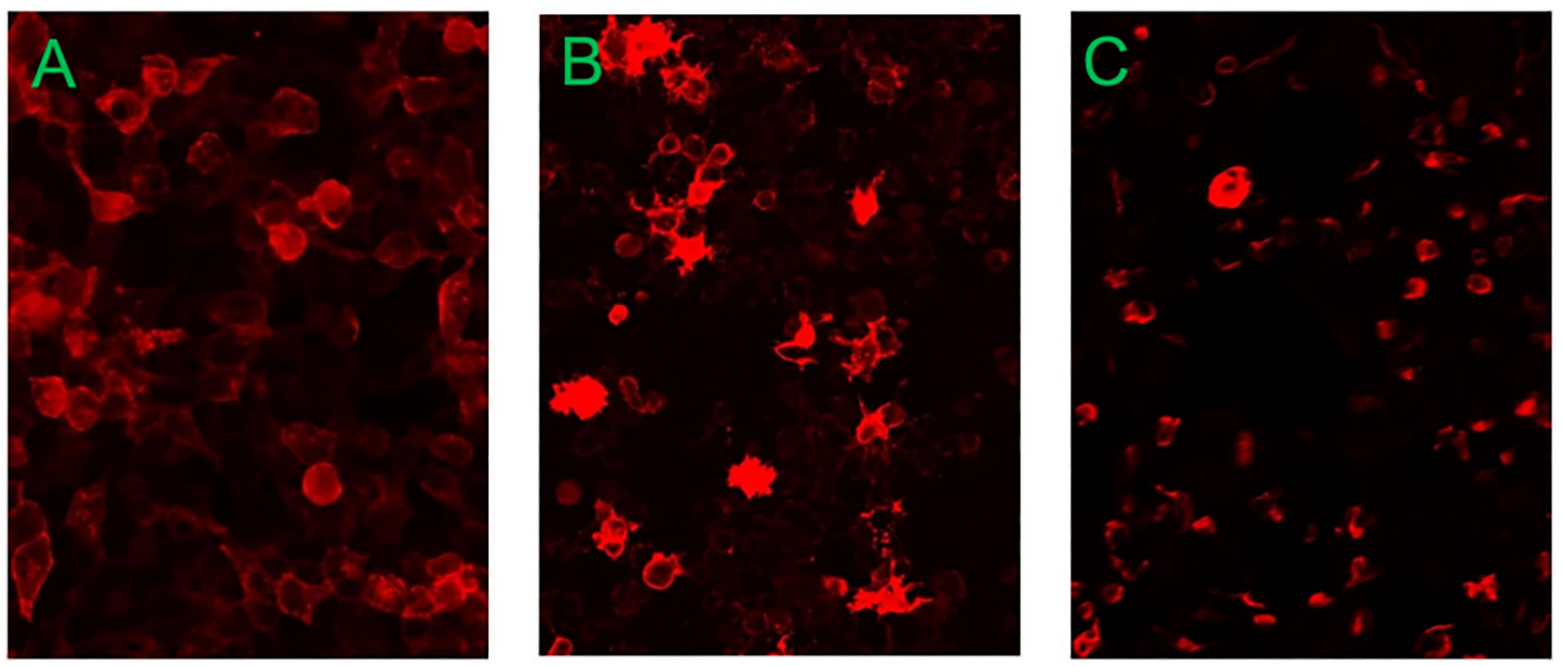
The results of CSF autoimmune encephalitis antibodies detected in Guangxi Golden Field Medical Laboratory were positive for anti–aquaporin-4 (AQP4) antibody (+; 1:10), positive for anti–glutamate receptor (NMDA-type) IgG antibody (+; 1:30), and positive for anti-GFAP antibody (+; 1:32). **(A)** Anti–aquaporin-4 antibody. **(B)** Anti–NMDA receptor antibody. **(C)** Anti-GFAP antibody.

The blood test showed a white blood cell count (WBC) of 15.16 × 10^9^/L. Cerebrospinal fluid (CSF) analysis suggested a WBC of 5.00 × 10^6^/L, CSF protein of 828.53 mg/L, and glucose of 2.83 mmol/L. CSF samples were sent to Guangxi Golden Field Medical Laboratory for testing of the twenty CSF autoimmune encephalitis antibodies through the cell-based assay. Its principle is to transfect the target antigen gene into mammalian cells to specifically express the corresponding antigen in mammalian cells. In addition, the green fluorescent protein was co-expressed during transfection as an internal reference for detection. Thereafter, the transfected cells were fixed onto the 96-well microplates and prepared into the antigen sheets. The specific antibodies in human serum, plasma, or CSF samples were detected semi-quantitatively by an indirect immunofluorescence method. The results were positive for anti-AQP4 antibody (+; 1:10), positive for anti–glutamate receptor (NMDA-type) Immunoglobulin G (IgG) antibody (+; 1:30), and positive for anti-GFAP antibody (+; 1:32) (**Figure 1**). The remaining autoimmune encephalitis antibodies including anti–glutamate receptor (AMPA1) antibody Immunoglobulin G (IgG), anti–glutamate receptor (AMPA2-type) antibody IgG, anti–leucine-rich glioma inactivated protein 1 (LGI 1) antibody IgG, anti–contact protein-associated protein 2 (CASPR2) antibody IgG, anti–gamma-aminobutyric acid–type B receptor (GABAB) antibody IgG, anti–Iglon family protein 5 (IgLON5) antibody IgG, anti–dipeptide similar protein 6 (DPPX) antibody IgG, anti–glycine receptor 1 (GlyR1) antibody IgG, anti–dopamine receptor 2 (DRD2) antibody IgG, anti–glutamate decarboxylase 2 (GAD65) antibody IgG, anti–metabolic glutamate receptor 5 (mGluR5) antibody IgG, anti–metabolic glutamate receptor 1 (mGluR1) antibody IgG, anti–synaptic protein-3α (Neurexin-3α) antibody IgG, anti–gamma-aminobutyric acid–type A receptor (GABAA) antibody, anti–Kelch-like protein 11 (KLHL11) antibody, anti–ganglionic acetylcholine receptor (ganglionic AChR) antibody, and anti–myelin oligodendrocyte glycoprotein antibody (MOG) were negative. Infectious disease screening by enzyme-linked immunosorbent assay suggested no abnormalities in hepatitis B surface antigen assay, hepatitis C antibody assay, human immunodeficiency virus antibody assay, treponema pallidum specific antibody assay, and syphilis non-specific antibody assay. Other laboratory tests, including antinuclear antibody assay, vasculitis test, folic acid, and vitamin B12, were normal. All other laboratory parameters were within normal limits.

**Figure 2:**
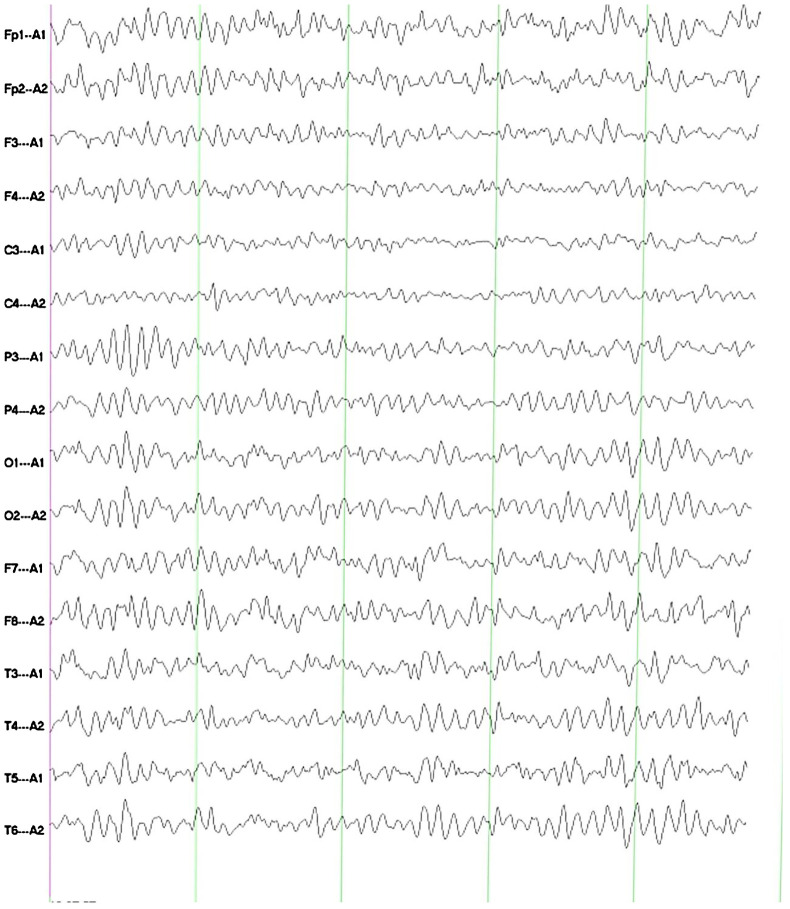
The EEG imaging indicates that, during awake and quiet closed-eye state, bilateral occipital regions exhibit predominant alpha rhythm activity at 9.0–10.0 Hz and 20–50 μV, with a basic symmetry between the left and right sides. Each lead shows minimal low-amplitude beta and theta waves, with widespread increase in alpha activity.

The electroencephalogram (EEG) imaging indicates that, during awake and quiet closed-eye state, bilateral occipital regions exhibit predominant alpha rhythm activity at 9.0–10.0 Hz and 20–50 μV, with a basic symmetry between the left and right sides. Each lead shows minimal low-amplitude beta and theta waves, with widespread increase in alpha activity (**Figure 2**).

**Figure 3:**
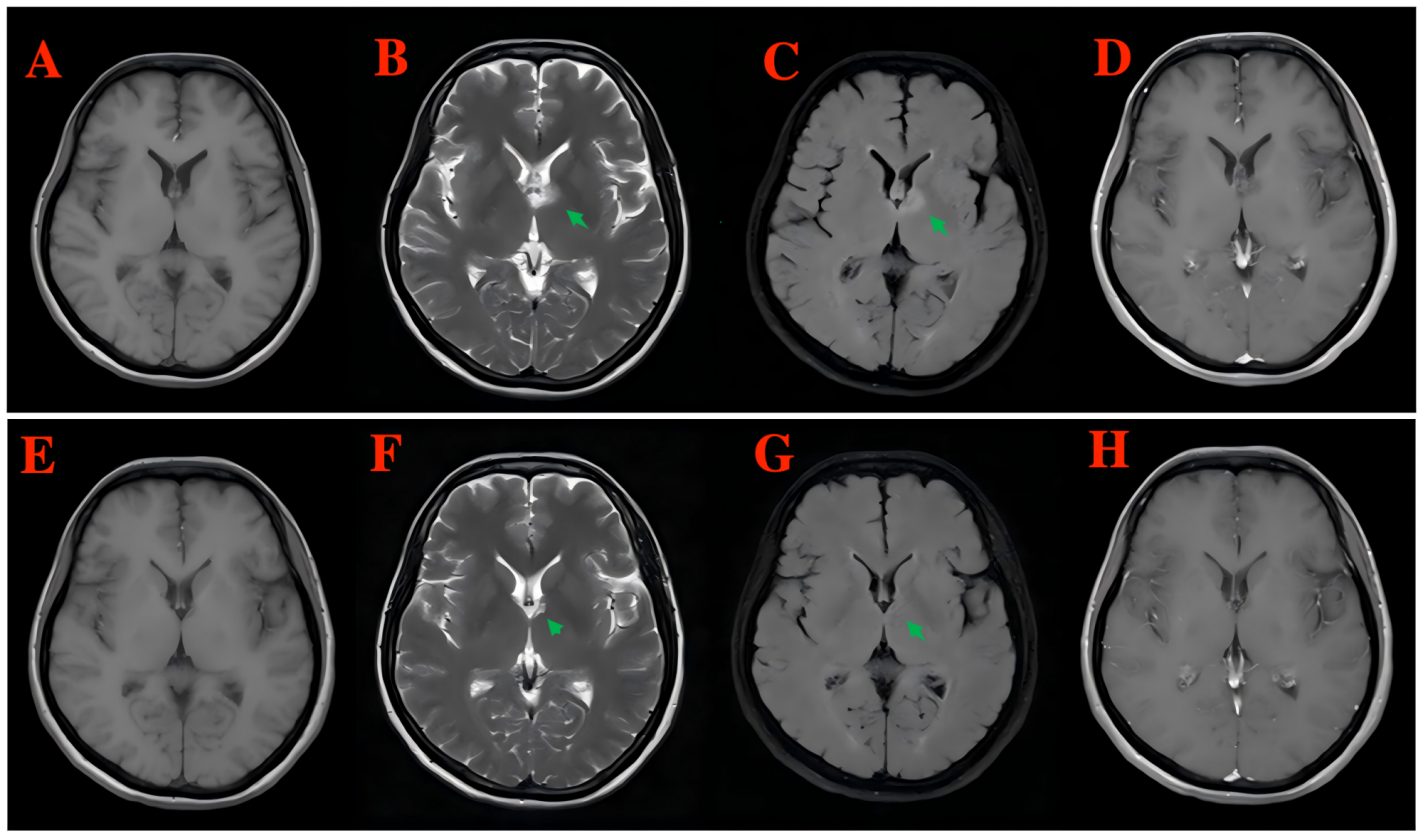
Brain MRI plain scan + enhancement with the gadolinium contrast agent revealed patchy hypointensity on T1WI, hyperintensity on T2WI, and hyperintensity on T2-FLAIR in the left basal ganglia, corona radiata, and local septum pellucida, with no enhancement in the enhanced lesions [**(A)** T1WI, **(B)** T2WI, **(C)** T2-FLAIR, and **(D)** enhanced T1-weighted MRI]. A follow-up MRI was performed 6 months later, which demonstrated the nearly total regression of the previous findings [**(E)** T1WI, **(F)** T2WI, **(G)** T2-FLAIR, and **(H)** enhanced T1-weighted MRI].

Brain magnetic resonance imaging (MRI) plain scan + enhancement with the gadolinium contrast agent revealed patchy hypointensity on T1WI, hyperintensity on T2WI, and hyperintensity on T2-FLAIR in the left basal ganglia, corona radiata, and local septum pellucida, with no enhancement in the enhanced lesions (**Figures 3A–D**). Spinal MRI showed no abnormality. A follow-up MRI was performed 6 months later, which demonstrated the nearly total regression of the previous findings (**Figures 3E–H**).

The patient received intravenous injection of methylprednisolone (500 mg/day for 5 days, consecutively), followed by a gradual dose reduction combined with mycophenolate mofetil (1 g, once daily) based on the hospital guidelines, and significant clinical and radiological improvements were observed. At present, the patient is still under follow-up, remains asymptomatic, and has no recurrence.

## Discussion

GFAP astrocytopathy is an inflammatory central nervous system disorder first reported and named by the Lennon team at the Mayo Clinic in the USA in 2016 (1). Currently, the epidemiology of GFAP astrocytopathy remains unclear, but, in a population-based comparative study on the incidence and prevalence of autoimmune and infectious encephalitis in Olmsted County, USA, the prevalence of GFAP astrocytopathy was found to be 0.0006% (0.6/100,000). In 2017, Flanagan et al. identified GFAP antibody targeting astrocytes as the specific biomarker for the disease (4). GFAP astrocytopathy has an acute or subacute onset, and some patients have predromal fever and a history of infection, with the main manifestations of symptoms of meningitis (headache and neck pain), encephalitis (delirium, tremors, seizures, or psychiatric symptoms), and myelitis (paresthesia and myasthenia), and optic neuritis (impaired loss) (5, 6). In this case, drowsiness was the only clinical manifestation of GFAP astrocytopathy, and this condition is rarely reported (7).

GFAP, as a type III intermediate filament protein, is expressed in cells and serves as a key component of the intermediates of mature astrocytes. GFAP not only functions as a biomarker for astrocytes but also participates in multiple biological processes, including maintenance of astrocyte morphology, blood-brain barrier, synaptic plasticity, regulation of cell proliferation, and transport of vesicles and lysosomes in astrocytes (8, 9). The pathogenesis of GFAP astrocytopathy has not been well understood yet, which may be related to the abnormal activation of the tumor necrosis factor pathway or GFAP peptide–specific CD8+ T lymphocytes, resulting in damage to the astrocytes (10). As shown in some studies, CSF examination in patients with GFAP astrocytopathy is dominated by inflammation, and most patients can experience elevated WBC and protein level (11). In some research, approximately 40% of patients with GFAP astrocytopathy have one or more co-existing neuronal autoantibodies, and multiple co-existing neuronal autoantibodies have been reported in some patients with GFAP astrocytopathy, with NMDAR antibody being the most common co-existing antibody, followed by AQP4 antibody (1, 4, 12). According to relevant literature report, patients first diagnosed with neuromyelitis optica spectrum disease positive for AQP4 develop GFAP antibody in their second attack and show typical clinical features of GFAP astrocytopathy (13). In this study, the patient did not have clinical symptoms, signs, or imaging evidence for a diagnosis of neuromyelitis optica spectrum disease and was positive for AQP4 antibody and NMDAR antibody, contributing to the diagnosis of this rare GFAP astrocytopathy positive for antibodies. GFAP antibody, AQP4 antibody, and NMDAR antibody are expressed in teratoma. When GFAP antibody combined with AQP4 antibody and NMDAR antibody is positive, the prediction rate is as high as 71% (14). However, we completed tumor marker testing and CT plain scan of the head, chest, and whole abdomen (including the pelvic cavity) and found no evidence of tumor. According to Flanagan et al., 35 out of 102 patients (34%) had tumors, and 66% of the tumors were detected within 2 years of symptom onset, including ovarian teratoma, adenocarcinoma, and glioma (4), which might be related to the short onset time of the disease and required long-term follow-up.

Currently, there are few research reports on the specific EEG changes associated with GFAP astrocytopathy. Some studies have reported that children with GFAP astrocytopathy may exhibit the EEG phenomenon known as extreme deltabrush (EDB) (15–18).Additionally, EDB has been identified as a specific EEG feature of NMDAR antibody encephalitis, although the underlying mechanisms remain unclear (19). Studies have indicated that patients with high antibody titers and the presence of EDB often present with more severe symptoms and may even experience conditions such as coma and status epilepticus, necessitating early intensive care (20). In this study, the patient’s EEG primarily showed minimal low-amplitude beta and theta waves in all leads, with widespread increase in alpha activity. EEG examination is convenient, non-invasive, and relatively cost-effective. The identification of abnormal EEG waveforms is of great significance for clinical diagnosis and disease assessment; thus, further research is warranted.

Head and spinal MRI is the preferred option of imaging examination for GFAP astrocytopathy. More than 50% of head and spinal MRI examinations for GFAP astrocytopathy show hypointensity on T1WI, whereas hyperintensity on T2WI and T2-FLAIR. The lesions are mainly located in the basal ganglia, followed by the thalamus, and most of them show bilateral symmetrical distribution. The typical head imaging feature is linear radial perivascular enhancement that is perpendicular to the ventricles (4, 20). In our case, although the lesions were located in the left basal ganglia, there was no typical imaging evidence of the disease.

For the time being, no unified diagnostic criteria have been developed for GFAP astrocytopathy, making the diagnosis particularly challenging. In our case, the presence of multiple antibodies, including AQP4, NMDAR, and GFAP, further complicates the diagnosis of primary GFAP astrocytopathy. Our case should be differentiated from non-convulsive status epilepticus (NCSE), viral encephalitis, and Wernicke’s encephalopathy (WE). For patients with persistent drowsiness, it is crucial to rule out NCSE, as it can present with altered consciousness without obvious convulsions. Long-term EEG monitoring is essential for its diagnosis (21–23). Additionally, central nervous system inflammation, such as viral encephalitis, particularly herpes simplex encephalitis (HSE), must also be excluded. HSE often presents with characteristic imaging findings, such as involvement of the temporal lobes and positive viral markers in CSF (24, 25).Furthermore, WE should be considered in the differential diagnosis. WE is a neurological disorder caused by thiamine (vitamin B1) deficiency and is typically characterized by a clinical triad: ocular motor dysfunction, ataxia, and mental status changes. Although the clinical presentation of WE can vary, the most common features include acute or subacute onset of altered mental status, ataxia, and nystagmus (26). Based on the patient’s clinical manifestations, physical examination findings, response to auxiliary investigations, and treatment outcomes, the final diagnosis was determined to be GFAP astrocytopathy with overlapping autoimmune syndrome. The patient responded well to the treatment regimen, showing significant clinical and radiological improvements following intravenous Methylprednisolone administration for 5 consecutive days, followed by a gradual dose reduction and mycophenolate mofetil therapy as per hospital guidelines.

The treatments for acute-phase GFAP astrocytopathy mainly include high-dose glucocorticoids (500–1,000 mg/day), intravenous injection of immunoglobulin, and plasma exchange therapy. Some patients require long-term oral administration of steroids and immunosuppressive agents such as mycophenolate mofetil, azathioprine, or rituximab to prevent disease recurrence (3, 5, 7, 12, 27). After active treatment, a majority of patients have a good prognosis, whereas some may develop disease recurrence, and a few may experience a variety of dysfunction or even death (5, 28).

## Conclusion

Currently, research regarding the diagnosis of GFAP astrocytopathy is scarce, and most physicians have insufficient understanding of the diagnosis of the diagnosis, which may easily lead to missed diagnosis and misdiagnosis. This case study adds to the limited research available on the presence of AQP4 antibody and NMDAR antibody in patients with GFAP astrocytopathy with overlapping positivity. Early detection and timely treatment are essential for the treatment of GFAP astrocytopathy, the improvement of long-term prognosis, and the reduction of mortality, which can thus avoid missed diagnosis and misdiagnosis and improve the clinical outcome of patients.

## Data Availability

The datasets presented in this article are not readily available because of ethical and privacy restrictions. Requests to access the datasets should be directed to the corresponding author.
